# Exposure to general anesthesia and risk of alzheimer's disease: a systematic review and meta-analysis

**DOI:** 10.1186/1471-2318-11-83

**Published:** 2011-12-14

**Authors:** Dallas P Seitz, Prakesh S Shah, Nathan Herrmann, Joseph Beyene, Naveed Siddiqui

**Affiliations:** 1 Department of Psychiatry, Queen's University, Kingston, Ontario, Canada; 2 Department of Pediatrics and Health Policy, Management, and Evaluation, University of Toronto, Toronto, Ontario, Canada; 3 Department of Psychiatry, University of Toronto and Sunnybrook Health Sciences Centre, Toronto, Ontario, Canada; 4 Department of Clinical Epidemiology and Biostatistics, McMaster University, Hamilton, Ontario, Canada; 5 Department of Anesthesia, Mount Sinai Hospital, Toronto, Ontario, Canada

**Keywords:** dementia, Alzheimer's disease, anesthesia, surgery, meta-analysis, systematic review

## Abstract

**Background:**

Alzheimer's disease (AD) is common among older adults and leads to significant disability. Volatile anesthetic gases administered during general anesthesia (GA) have been hypothesized to be a risk factor for the development of AD. The objective of this study is to systematically review the association between exposure to GA and risk of AD.

**Methods:**

We searched electronic databases including MEDLINE, Embase, and Google scholar for observational studies examining the association between exposure to GA and risk of AD. We examined study quality using a modified version of the Newcastle-Ottawa risk of bias assessment for observational studies. We used standard meta-analytic techniques to estimate pooled odds ratios (OR) and 95% confidence intervals (CI). Subgroup and sensitivity analyses were undertaken to evaluate the robustness of the findings.

**Results:**

A total of 15 case-control studies were included in the review. No cohort studies were identified that met inclusion criteria. There was variation in the methodological quality of included studies. There was no significant association between any exposure to GA and risk of AD (pooled OR: 1.05; 95% CI: 0.93 - 1.19, Z = 0.80, *p *= 0.43). There was also no significant association between GA and risk of AD in several subgroup and sensitivity analyses.

**Conclusions:**

A history of exposure to GA is not associated with an increased risk of AD although there are few high-quality studies in this area. Prospective cohort studies with long-term follow-up or randomized controlled trials are required to further understand the association between GA and AD.

## Background

Alzheimer's disease (AD) and related forms of dementia affect 13% of all adults over age 65 and the number of individuals with dementia in the United States is expected to increase from a current prevalence of 5.1 million to over 7.7 million individuals by 2030[1]. The effects of AD on society are substantial. In the United States the annual direct costs associated with AD are $148 billion and caregivers provide 8.5 billion hours of care annually[1]. Alzheimer's disease is also a leading reason for admission to long-term care facilities.

The pathophysiology of AD involves the accumulation of protein plaques composed of β-amyloid and the formation of neurofibrillary tangles related to hyperphosphorylation of tau proteins[2]. The cause of AD is multi-factorial with some of the strongest risk factors for AD include advanced age, female gender, educational achievement, family history and specific genetic mutations[3]. There are few modifiable risk factors which have been identified for AD, which include a history of head trauma and most cardiovascular risk factors[3].

Short-term cognitive dysfunction lasting days to weeks has been commonly observed following surgery[4] and is often referred to as postoperative cognitive dysfunction (POCD). Recently, some potential mechanisms linking inhaled volatile anesthetics to AD pathology have been proposed to provide a link between exposure to general anesthesia (GA) and the subsequent development of POCD or AD following surgery[5]. Commonly utilized inhaled anesthetics have been demonstrated to increase the formation of AD precursors including β-amyloid plaques[6,7], and neurofibrillary tangles[8,9] in animal models and *in vitro *studies. However, randomized controlled trials have failed to demonstrate an increased risk of persistent cognitive impairment in the 1 - 2 years following exposure to GA when compared to regional anesthesia[10]. To date there have been no randomized controlled trials that have examined the risk of developing cognitive impairment meeting clinical criteria for AD or related forms of dementia associated with exposure to GA. There have been previous narrative reviews on the potential relationship between anesthesia and POCD[11,12] and systematic reviews which have examined the relationship between GA and postoperative delirium or POCD[13-17]. Most reviews found that there was limited evidence to suggest any difference between GA anesthesia and regional anesthesia on the incidence of post-operative delirium or POCD [13,15-17]. One exception was a review on anesthesia for hip fracture surgery which found a reduction in acute postoperative confusion for regional anesthesia compared to GA [14]. To date there are no reviews that have examined whether exposure to GA is a risk factor for the development of AD. Therefore, the objective of our study is to systematically review the literature on observational studies examining the association between exposure to GA and subsequent development of AD. Understanding the risk of AD associated with exposure to GA would help in determining the relationship between GA and AD and inform strategies to prevent or minimize the risk of AD following surgical procedures.

## Methods

### Search strategy

We searched MEDLINE, EMBASE, and Google Scholar using key words and medical subject headings to identify relevant articles (see Additional File 1, Document 1). The reference lists of retrieved articles were hand-searched for additional references. There were no language restrictions and data from both published and unpublished studies were included provided that sufficient information was available for data extraction. Two authors (DS and NS) were involved in the selection of studies for the review and discrepancies were resolved by discussion after retrieving the full text of articles in question.

### Types of studies

We followed the MOOSE[18] and PRISMA[19] guidelines for the reporting of meta-analysis of observational studies. Observational studies examining exposure to GA and risk of AD including both case-control and cohort studies were eligible for the review. We included human studies where the mean age of study population was ≥ 50 years of age. We excluded studies that examined POCD, postoperative delirium, or abnormalities on neuropsychological testing without a diagnosis of dementia from the review to focus only on the outcome of development of AD.

### Definition of exposure

Exposure to GA was defined as any reported history of surgery under GA when compared to no history of surgery under GA. Where information was available, the following information was also recorded: history of exposure to GA when compared to regional anesthesia (RA); the number of previous GAs; cumulative exposure to GA as measured in minutes of GA exposure; the type of surgery performed under GA (cardiovascular, non-cardiovascular, or neurological); the type of agent utilized for GA; and, duration of time between exposure to GA and assessment for AD.

### Definition of Outcomes

The primary outcome was a diagnosis of AD of any severity according to clinical impression or standard AD diagnostic criteria. These AD criteria included: National Institutes of Neurological and Communicative Disorders and Stroke - Alzheimer's Disease and Related Disorders (NINDS-ADRDA)[20]; Diagnostic and Statistical Manual of Mental Disorders (DSM)[21]; or the *International Classification of Diseases*. Secondary outcomes included time to development of AD, and the development of early-onset (<65 years) or late-onset AD (>65 years).

### Data extraction

Two reviewers (DS and NS) extracted data from included studies using a standard data extraction form. Information extracted from studies included: age of participants, gender distribution, source population and description of sample selection methods. We also recorded potential confounders including medical morbidity and indication for surgery. For case-control studies, we recorded the number of cases and controls with exposure to GA and the summary odds ratio (OR) and 95% confidence intervals (CI) reported in studies from matched or adjusted analyses.

### Assessment of Study Quality

Primary studies were reviewed according to the STROBE criteria[22] and study quality checklist based on the Newcastle-Ottawa criteria for case-control and cohort studies was developed[23] to describe potential risk of bias according to seven aspects of study quality (see Additional File 2, Document 2). We classified case-control studies as being at overall high or low risk of bias. A case-control study was considered to be at low risk of bias if all of the following study quality items were recorded as low risk of bias: definition of cases, sample used for selection of controls, and matching or adjusting for a minimum of age and gender.

### Data Synthesis and Meta-Analysis

We included a qualitative description of studies meeting inclusion criteria using text and tables. Studies were first assessed qualitatively for homogeneity and homogeneous studies were combined in meta-analysis to arrive at (OR) and 95% confidence intervals (CI) for the association between GA and AD. We combined case-control studies that controlled for a minimum of both age and gender either through matching or statistical adjustment. When multiple control groups were used in a study we only included the control group that was most representative of the source population for meta-analysis. Random effects models were utilized for all meta-analyses given the expected heterogeneity between different studies. Study weights were assigned using the inverse of the study variance. The software package Comprehensive Meta-Analysis (version 2.2) was utilized for all analyses.

### Heterogeneity

We assessed study heterogeneity qualitatively by assessing study populations and study designs. We assessed statistical heterogeneity quantitatively using the Cochran Q statistic and used p-values of ≤0.1 as our threshold for heterogeneity. We used the I^2 ^statistic to quantify the degree of statistical heterogeneity.

### Subgroup and Sensitivity Analysis

We planned to analyse case-control and cohort studies were separately. We planned to undertake the following subgroup analyses: by control group (no surgery control groups or regional anesthesia control groups); high and low risk of bias studies; AD diagnosis (standard AD criteria compared to clinical criteria or other methods of diagnosis); and, type of surgery (non-cardiac surgery, neurological, or cardiac surgery). We also examined the summary OR for exposure to any GA after excluding each study sequentially to evaluate for studies that may have had a large influence on the results of the meta-analysis. We also conducted a meta-regression of the association between the reported OR for GA exposure and risk of AD using year of publication as an independent variable as older anesthestic agents may be associated with a greater risk of AD when compared to newer anesthetics[6].

### Publication Bias

We assessed the potential publication bias by visual inspection of the funnel plot produced by plotting the standard error against the log OR of studies.

## Results

### Study Selection

**Figure 1:**
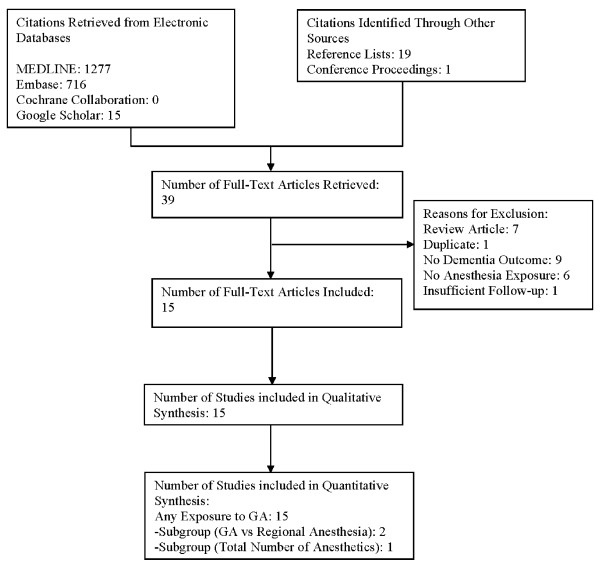
**Flow of Studies through Review Process**.

The flow of studies through the review process is outlined in Figure 1. A total of 15 case-control studies were identified that met inclusion criteria[24-38]. Fourteen of the included studies were published as full manuscripts and one was presented in poster form at a conference[37]. There were no cohort studies that met our inclusion criteria.

### Description of Included Studies

The characteristics of the included studies are described in (see Additional File 3, Table S1). A total of 1,752 cases and 5,261 controls were included in the respective studies. Cases and controls in most studies were comparable in terms of age and gender distribution. Ten studies used matching to control for potential confounding related to age and gender with the remaining 5 studies using statistical adjustment for these factors. Eight studies determined exposure to GA through interviews with proxies of cases, and 5 studies used medical records to determine exposure. Two studies used incident cases of dementia from cohort studies and exposure to GA was determined through interviews of individuals prior to them developing AD.

### Assessment of Risk of Bias

The potential risk of bias associated with various aspects of study design is described in Additional File 4, Table S2. Four studies were rated as being at overall low risk of bias[32,33,36,37] with the remaining studies being at higher potential risk of bias due to some aspect of study design.

### Meta-Analysis

#### Association between Exposure to Any Surgery with General Anesthetic and Alzheimer's Disease

**Figure 2:**
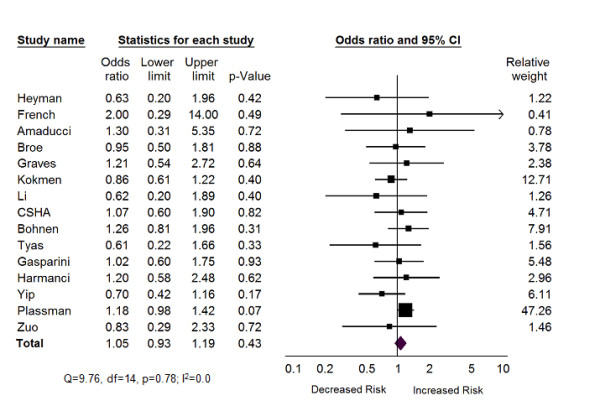
**Forest Plot of Any Exposure to General Anesthesia and Alzheimer's Disease**.

All of the 15 studies included in the systematic review controlled for age and gender and were subsequently suitable to combine in meta-analysis. The association between exposure to GA and risk of AD for each of the studies is summarized in Additional File 5, Table S3. Meta-analysis of the 15 studies revealed no statistically significant association between previous exposure to GA and development of AD (pooled OR = 1.05; 95% CI: 0.93 - 1.19: Z = 0.80, *p *= 0.43) (Figure 2). There was little evidence of statistical heterogeneity between studies as assessed by the Q statistic (Q = 9.76, df = 14, p = 0.78) and the magnitude of statistical heterogeneity was minimal (I^2 ^= 0.0%).

### Subgroup Analysis

#### Exposure to General Anesthesia Compared to Regional Anesthesia

**Figure 3:**
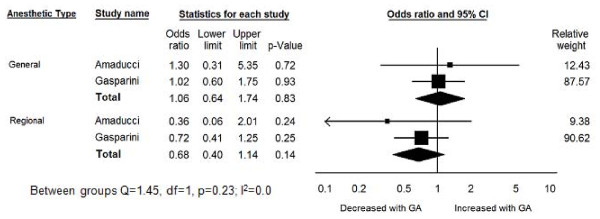
**Forest Plot of General Anesthesia Compared to Regional Anesthesia and Risk of Alzheimer's Disease**.

A total of two studies reported on the risk of AD associated with GA and regional anesthesia[26,30]. The OR for AD associated with exposure to GA was 1.06 (95% CI: 0.64 -1.74, p = 0.83) in these two studies (Figure 3). The OR for AD associated with previous exposure to regional anesthesia was 0.68 (95% CI: 0.4 - 1.14, *p *= 0.14). The difference in the odds ratios was not statistically significant (Q = 0.55, df = 1, *p *= 0.46).

#### Number of Prior Anesthetics or Cumulative Exposure to General Anesthesia

One study reported on the association between number of anesthetics and the cumulative duration of anesthesia and AD[31]. There was no statistically significant association between the number of surgeries involving GA or cumulative exposure to GA. The OR for exposure to six or more surgeries under general compared to fewer than six was 1.44 (95% CI: 0.77 - 2.71) and the association between a cumulative duration of greater than 10 hours of exposure to GA when compared to less than 10 hours was 1.63 (95% CI: 0.53 - 5.04).

#### Studies with Low Risk of Bias Compared to High Risk of Bias

A total of four studies [32,33,36,37] were rated as being at overall low risk of bias according to our risk of bias assessment. The OR for exposure to GA and risk of AD in these low risk of bias studies was 1.09 (95% CI: 0.93 - 1.28; Z = 1.05, p = 0.29) while the OR in the high risk of bias studies was 1.00 (95%CI: 0.82 - 1.21; Z = 0.03, p = 0.98). There was no statistically significant difference in the OR between the high and low risk of bias studies (Q = 0.83, df = 1, p = 0.36).

#### Criteria for Defining Dementia

Eight studies used standardized criteria for diagnosing AD [27,28,30-35] and seven studies used clinical criteria or other methods for diagnosing AD [24-26,29,36,38,39]. The OR for exposure to GA and development of AD in studies that used standard criteria was 1.06 (95% CI: 0.84 - 1.33, p = 0.63) and the OR for GA and AD in the remaining studies was 1.05 (95% CI: 0.90 - 1.22, p = 0.53). There was no significant difference in the OR for these subgroups (Q = 0.13, df = 1, p = 0.72.).

#### Type of Surgery

There were no studies included in our review that specified the type of surgery that was performed under general anesthesia so no subgroup analyses could be undertaken.

#### Time Between Exposure to General Anesthesia and Assessment of Outcome

Only one study [25] specified a minimum duration of 5 years between exposure to GA and AD with an OR of 0.61 (0.22 - 1.63) while the remaining studies did not report the duration of time elapsed between GA exposure and assessment of dementia.

#### Sensitivity Analysis and Meta-Regression

The OR for exposure to any GA and AD was not statistically significant when calculated after each study was sequentially excluded. In addition, the summary OR did not change after excluding the two studies that used stepwise regression to adjust for confounders[35,38] when compared to studies that explicitly matched or adjusted for a minimum of age and gender.

There was no significant association observed between the log OR and year of publication in meta-regression. To investigate the potential effect that additional studies would have had on our results, we calculated the Orwin's failsafe N to determine the number of studies of a given effect size that would be required to change the observed OR by a given amount. We selected a mean observed OR for a hypothetical group of missing studies to be 1.3 based on the observed upper range of the ORs in the studies identified in our review. Assuming a minimal clinically significant OR of 1.2 for the association between GA and AD, an additional 25 studies, each with a mean observed OR of 1.3, would be needed to arrive at an statistically significant OR of 1.2.

### Publication Bias

There was no evidence of potential publication bias as assessed by visual inspection of the funnel plot produced by plotting the standard error against the log OR of studies.

## Discussion

This is the first systematic review and meta-analysis of observational studies examining the association between exposure to GA and risk of developing AD. We found a number of case-control studies and no cohort studies that examined the association between GA and development of AD. Meta-analysis did not reveal any association between previous exposure to GA and development of AD. This finding was supported by several subgroup and sensitivity analyses. However, many of the studies included in our review were at some potential risk of bias from certain aspects of study design and the potential biases inherent in all observational studies may have contributed to the observed findings.

The findings from our review are consistent with the existing clinical literature on the lack of long-term cognitive sequelae associated with exposure to GA. Although cognitive dysfunction is common following surgery, much of the observed changes in cognition may be attributed to the stress of surgery and recovery[12,39] rather than any specific effects of anesthetic technique. Large-scale studies have reported that the prevalence of POCD in the week following non-cardiac surgery is 25% with 9.9% of individuals continuing to display cognitive dysfunction at 3 months following surgery[4]. However, a randomized controlled trial comparing GA to regional anesthesia in non-cardiac surgery found little difference in persistent POCD associated with different anesthetic techniques. In this study, regional anesthesia was associated with a non-statistically significant difference in early POCD at day 7 (19.7% vs 12.5%), although the proportions of individuals with POCD in the regional and GA groups were similar at 3 months (14.3% vs. 13.9%)[40]. This same study reported that most individuals recovered cognitive function by 1 - 2 years following surgery[10], indicating that there is little evidence of long-term cognitive consequences associated with GA when compared to regional techniques. Similar results have been reported in other randomized controlled trials comparing GA to regional anesthesia in the elderly[41] as well as observational studies[39]. Systematic reviews of POCD following non-cardiac surgery identified limited evidence for long-term cognitive effects of GA although there were relatively few well-designed studies with follow-up times of sufficient duration to allow for the development of AD following exposure to anesthesia[13,17]. POCD appears to be more common following cardiac surgery when compared to non-cardiac surgery likely due to factors such as cardiopulmonary bypass, intraoperative hypotension, and risk factors which are common for both cardiovascular disease and cerebrovascular disease[42,43]. The relative contribution of GA to POCD in cardiac surgery setting is difficult to ascertain as many cardiac procedures cannot be undertaken using regional anesthetic techniques.

In contrast to our review and previous prospective studies, evidence from animal models and *in vitro *studies suggesting that exposure to GA may promote AD processes. Animal studies have suggested that exposure to the volatile anesthetic gas halothane may increase amyloid deposition in transgenic mice while isoflurane had little effect on amyloid deposition[6]. Another animal study found that sevoflurane was also associated with increases in beta-amyloid in mouse models[44]. Increases in tau hyperphosphorylation have also been observed with exposure to ether anesthesia[8]. Additional human tissue culture studies report that isoflurane[7,45,46], desflurane[47], and sevoflurane[7] may also be associated with increases in beta-amyloid related pathology. In our review we were not able to ascertain possible differential effects of individual anesthetic agents. However, halothane is an older medication that has largely been replaced by newer anesthetic agents and our study did not find that there was any association between year of publication and association between GA and AD. To date, there have been no studies that have examined the effects of GA on AD pathology in humans following typical exposure to GA through either biomarkers or neuroimaging and the results observed in these basic studies require further confirmation from human studies to determine the clinical significance of these findings.

The main strengths of our study include the rigorous methods employed to identify studies and assessment of potential risk of bias. The lack of association between GA and risk of AD was robust to a number of subgroup and sensitivity analyses. We did not observe any evidence of publication bias in the studies included in our review, which decreases the likelihood that our findings were related to our method of selecting articles. Finally, the observed lack of association between exposure to GA and AD is not likely to be affected by confounding related to factors associated with requiring a surgical procedure as this would have been expected to show an elevated risk of AD associated with GA which was not observed.

There are potential limitations to our study. Recall and information bias are problematic in all case-control studies and are potentially of greater importance in case-control studies of conditions such as AD where individuals cannot provide an accurate record of past exposures. Other studies have shown that proxy reporters can be used to determine exposure status in case-control studies of neurological diseases although the accuracy of reporting is dependent on the nature of the exposure[48]. All of the studies included in our review attempted to address this source of bias by using proxy reporters for exposure history or using medical records. In three of the studies included in this review, the accuracy of proxy reporters for determining exposure history was assessed through agreement between the history provided by controls with normal cognition and proxy reporters of these controls[24,28,32] with agreement between controls and proxies of controls on history of exposure to GA in the moderate range of agreement indicating that proxy reports of exposure may be prone to bias. Other important factors such as potential dose-response relationship were only available from one study and no studies included information on the type of surgery which may have an important impact on the subsequent risk of AD. Finally, some individuals with early cognitive impairment or dementia may be less likely to be offered surgical procedures which may have reduced the apparent observed risk of AD associated with GA in our review.

## Conclusions

At the present time there we found no evidence to support an association between exposure to GA and increased risk of developing AD based on available observational studies. Patients should be warned about the potential long-term cognitive sequelae of undergoing surgical procedures and efforts should be made to optimize the perioperative care of older adults who are at risk of delirium and postoperative cognitive decline. The decision to use GA over other anesthetic techniques should be made on the basis of the surgical procedure and other clinical factors related to anesthetic choice. Further long-term prospective cohort studies or randomized controlled trials using biomarkers or neuroimaging modalities are required to further understand the associations between GA and AD.

## Abbreviations

AD: Alzheimer's disease; CI: confidence interval; GA: general anesthesia; OR: odds ratio; POCD: postoperative cognitive decline; RA: regional anesthesia.

## Competing interests

The authors declare that they have no competing interests.

## Authors' contributions

DS and NS completed the literature search, data extraction, data analysis and drafted the manuscript. PS contributed to the study conception, data analysis and revising the manuscript. NH contributed to the study conception, data analysis, data interpretation and revising the manuscript. JB contributed to the data analysis and interpretation of data, and revising the manuscript. All authors have given final approval to the version being published.

## Pre-publication history

The pre-publication history for this paper can be accessed here:

http://www.biomedcentral.com/1471-2318/11/83/prepub

## Supplementary Material

Additional file 1 **Document 1: Electronic Database Search Strategy**. Click here for file

Additional file 2 **Document 2: Risk of Bias Assessment Tool - Adapted from the Newcastle-Ottawa Risk of Bias Assessment for Case-Control Studies**. Click here for file

Additional file 3 **Table S1: Case-Control Studies Examining Association between General Anesthesia and Alzheimer's Disease**. Click here for file

Additional file 4 **Table S2: Association between Exposure to General Anesthesia and Alzheimer's Disease in Case-Control Studies**. Click here for file

Additional file 5 **Table S3: Assessment of Risk of Bias in Included Studies**. Click here for file

## References

[B1] Alzheimer Association 2009 Alzheimer's disease facts and figures Alzheimer Dement 2009 5 234 270 10.1016/j.jalz.2009.03.001 19426951

[B2] Querforth HW LaFerla FM Alzheimer's Disease Mechanisms of Disease N Engl J Med 2010 362 329 344 10.1056/NEJMra0909142 20107219

[B3] Patterson C Feightner JW Garcia A Hsiung GY MacKnight C Sadovnick AD Diagnosis and treatment of dementia: 1. Risk assessment and primary prevention of Alzheimer disease CMAJ 2008 178 48 56 10.1503/cmaj.070796 PMC2244657 18299540

[B4] Moller JT Cluitmans P Rasmussen LS Houx P Rasmussen H Canet J Rabbit P Jolles J Larsen K Hanning CD Langeron O Johnson T Lauven PM Kristensen PA Biedler A van Beem H Fraidakis O Silverstein JH Beneken JE Gravenstein JS Long-term postoperative cognitive dysfunction in the elderly ISPOCD1 study. ISPOCD investigators. International Study of Post-Operative Cognitive Dysfunction Lancet 1998 351 857 61 10.1016/S0140-6736(97)07382-0 9525362

[B5] Bilotta F Doronzio A Stazi E Titi L Fodale V Di Nino G Rosa G Postoperative cognitive dysfunction: toward the Alzheimer's disease pathomechanism hypothesis J Alzheimers Dis 2010 22 Suppl 3 81 9 20930308 10.3233/JAD-2010-100825

[B6] Bianchi SL Tran T Liu C Lin S Li Y Keller JM Eckenhoff RG Eckenhoff MF Brain and behavior changes in 12-month-old Tg2576 and nontransgenic mice exposed to anesthetics Neurobiol Aging 2008 29 1002 10 10.1016/j.neurobiolaging.2007.02.009 17346857 PMC4899817

[B7] Xie Z Dong Y Maeda U Alfille P Cully DJ Crosby G Tanzi RE The common inhalation anesthetic isoflurane induces apoptosis and increases amyloid beta protein levels Anesthesiology 2006 104 988 94 10.1097/00000542-200605000-00015 16645451

[B8] Ikeda Y Ishiguro K Fujita SC Ikeda Y Ishiguro K Fujita S Ether stress-induced Alzheimer-like tau phosphorylation in the normal mouse brain FEBS Letters 2007 581 891 7 10.1016/j.febslet.2007.01.064 17289030

[B9] Planel E Richter KE Nolan CE Finley JE Liu L Wen Y Krishnamurthy P Herman M Wang L Schachter JB Nelson RB Lau LF Duff KE Anesthesia leads to tau hyperphosphorylation through inhibition of phosphatase activity by hypothermia J Neurosci 2007 27 3090 7 10.1523/JNEUROSCI.4854-06.2007 17376970 PMC6672474

[B10] Abildstrom H Rasmussen LS Rentowl P Hanning CD Rasmussen H Kristensen PA Moller JT Cognitive dysfunction 1-2 years after non-cardiac surgery in the elderly. ISPOCD group. International Study of Post-Operative Cognitive Dysfunction Acta Anaesthesiol Scand 2000 44 1246 51 10.1034/j.1399-6576.2000.441010.x 11065205

[B11] Baranov D Bickler PE Crosby GJ Culley DJ Eckenhoff MF Eckenhoff RG Hogan KJ Jevtovic-Todorovic V Palotas A Perouansky M Planel E Silverstein JH Wei H Whittington RA Xie Z Zuo Z Consensus statement: First International Workshop on Anesthetics and Alzheimer's disease Anesth Analg 2009 108 1627 30 10.1213/ane.0b013e318199dc72 19372347 PMC2769511

[B12] Cryns AG Gorey KM Goldstein MZ Effects of surgery on the mental status of older persons. A meta-analytic review J Geriatr Psychiatry Neurol 1990 3 184 91 10.1177/089198879000300402 2149929

[B13] Newman S Stygall J Hirani S Shaefi S Maze M Postoperative cognitive dysfunction after noncardiac surgery: a systematic review Anesthesiology 2007 106 572 90 10.1097/00000542-200703000-00023 17325517

[B14] Parker MJ Handoll HHG Griffiths R Anaesthesia for hip fracture surgery in adults Cochrane Database Syst Rev 2009 4 CD000521 10.1002/14651858.CD000521 11687085

[B15] Bryson GL Wyand A Evidence-based clinical update: general anesthesia and the risk of delirium and postoperative cognitive dysfunction Can J Anaesth 2006 53 669 77 10.1007/BF03021625 16803914

[B16] Wu CL Hsu W Richman JM Raja SN Postoperative cognitive function as an outcome of regional anesthesia and analgesia Reg Anesth Pain Med 2004 29 257 68 15138912 10.1016/j.rapm.2003.11.007

[B17] Mason SE Noel-Storr A Ritchie CW The impact of general and regional anesthesia on the incidence of post-operative cognitive dysfunction and post-operative delirium: a systematic review with meta-analysis J Alzheimers Dis 2010 22 Suppl 3 67 79 20858956 10.3233/JAD-2010-101086

[B18] Stroup DF Berlin JA Morton SC Olkin I Williamson GD Rennie D Moher D Becker BJ Sipe TA Thacker SB Meta-Analysis of Observational Studies in Epidemiology: A Proposal for Reporting JAMA 2000 283 2008 2012 10.1001/jama.283.15.2008 10789670

[B19] Moher D Liberati A Tetzlaff J Altman DG PRISMA Group Preferred Reporting Items for Systematic Reviews and Meta-Analyses: The PRISMA Statement PLOS Med 2009 6 e1000097 10.1371/journal.pmed.1000097 19621072 PMC2707599

[B20] McKhann G Drachman D Folstein M Katzman R Price D Stadlan EM Clinical diagnosis of Alzheimer's disease: report of the NINCDS-ADRDA Work Group under the auspices of Department of Health and Human Services Task Force on Alzheimer's Disease Neurology 1984 34 939 944 6610841 10.1212/wnl.34.7.939

[B21] American Psychiatric Association Arlington VA Diagnostic and Statistical Manual of Mental Disorders 2000 Fourth American Psychiatric Association Text Revision

[B22] von Elm E Altman D Egger M Pocock SJ Gotzsche PC Vandenbrouke JP The Strengthening of Reporting of Observational Studies in Epidemiology Statement: Guidelines for Reporting Observational Studies PLoS Med 2007 4 e296 10.1371/journal.pmed.0040296 17941714 PMC2020495

[B23] Ottawa Health Research Institute The Newcastle-Ottawa Scale (NOS) for assessing the quality of non-randomized studies in meta-analysis http://www.ohri.ca/programs/clinical_epidemiology/oxford.htm

[B24] Heyman A Wilkinson WE Stafford JA Helms MJ Sigmon AH Weinberg Alzheimer's Disease: A Study Epidemiological Aspects Ann Neurol 1984 15 335 341 10.1002/ana.410150406 6742780

[B25] French LR Schuman LM Mortimer JA Hutton JT Boatman RA Christians B A case-control study of dementia of the Alzheimer type Am J Epidemiol 1985 21 414 21 10.1093/oxfordjournals.aje.a114013 4014131

[B26] Amaducci LA Fratiglioni L Rocca WA Fieschi C Livrea P Pedone D Bracco L Lippi A Risk factors for clinically diagnosed Alzheimer's disease: a case-control study of an Italian population Neurology 1986 36 922 31 3714054 10.1212/wnl.36.7.922

[B27] Broe GA Henderson SA Creasey H McCusker E Korten AE Jorm AF Longley W Anthony JC A case-control study of Alzheimer's disease in Australia Neurology 1990 40 1698 707 2146525 10.1212/wnl.40.11.1698

[B28] Graves AB White E Koepsell TD Reifler BV van Belle G Larson EB Raskind M A case-control study of Alzheimer's disease Ann Neurol 1990 28 766 74 10.1002/ana.410280607 2285263

[B29] Kokmen E Beard CM Chandra V Offord KP Schoenberg BS Ballard DJ Clinical risk factors for Alzheimer's disease: a population-based case-control study Neurology 1991 41 1393 7 1891088 10.1212/wnl.41.9.1393

[B30] Li G Shen YC Li YT Chen CH Zhau YW Silverman JM A case-control study of Alzheimer's disease in China Neurology 1992 42 1481 8 1641140 10.1212/wnl.42.8.1481

[B31] Bohnen NI Warner MA Kokmen E Beard CM Kurland LT Alzheimer's disease and cumulative exposure to anesthesia: a case-control study J Am Geriatr Soc 1994 42 198 201 8126336 10.1111/j.1532-5415.1994.tb04952.x

[B32] The Canadian Study of Health and Aging: The Canadian Study of Health and Aging Risk factors for Alzheimer's disease in Canada Neurology 1994 44 2073 2080 7969962 10.1212/wnl.44.11.2073

[B33] Tyas SL Manfreda J Strain LA Montgomery PR Risk factors for Alzheimer's disease: a population-based, longitudinal study in Manitoba, Canada Int J Epidemiol 2001 30 590 7 10.1093/ije/30.3.590 11416089

[B34] Gasparini M Vanacore N Schiaffini C Brusa L Panella M Talarico G Bruno G Meco G Lenzi GL Neurolog A case-control study on Alzheimer's disease and exposure to anesthesia Neurol Sci 2002 23 11 4 10.1007/s100720200017 12111615

[B35] Harmanci H Emre M Gurvit H Bilgic B Hanagasi H Gurol E Sahin H Tinaz S Risk factors for Alzheimer disease: a population-based case-control study in Istanbul, Turkey Alzheimer Dis Assoc Disord 2003 17 139 45 10.1097/00002093-200307000-00003 14512826

[B36] Yip AG Brayne C Matthews FE Risk factors for incident dementia in England and Wales: The Medical Research Council Cognitive Function and Ageing Study. A population-based nested case-control study Age Ageing 2006 35 154 60 16414964 10.1093/ageing/afj030

[B37] Plassman BL Langa KM Finlayson EVA Rogers MAM Surgery using general anesthesia and risk of dementi in the Aging, Demographics and Memory Study, in *I*nternational Conference on Alzheimer's Dementia 2009

[B38] Zuo C Zuo Z Spine Surgery under General Anesthesia May Not Increase the Risk of Alzheimer's Disease Dement Geriatr Cogn Disord 2010 29 233 239 10.1159/000295114 20375503 PMC2865396

[B39] Ancelin M-L de Roquefeuil G Scali J Bonnel J Adam JF Cheminal JC Cristol JP Dupuy A-M Carriere I Ritchie K Long-term post-operative cognitive decline in the elderly: the effects of anesthesia type, apolipoprotein E genotype, and clinical antecedents J Alzheimers Dis 2010 22 Suppl 3 105 13 20858969 10.3233/JAD-2010-100807 PMC3078520

[B40] Rasmussen LS Johnson T Kuipers HM Kristensen D Siersma VD Vila P Jolles J Papioannou A Abildstrom H Silverstein JH Bonal JH Raeder J Nielson IK Korttila K Munoz L Dodds C Moller JT Investigators IPOCD Does anaesthesia cause postoperative cognitive dysfunction? A randomised study of regional versus general anaesthesia in 438 elderly patients Acta Anaesthesiol Scand 2003 47 260 6 10.1034/j.1399-6576.2003.00057.x 12648190

[B41] Nielson WR Gelb AW Casey JE Penny FJ Merchant RN Manninen PH Long-term cognitive and social sequelae of general versus regional anesthesia during arthroplasty in the elderly Anesthesiology 1990 73 1103 9 10.1097/00000542-199012000-00006 2248389

[B42] Gao L Taha R Gauvin D Othmen LB Wang Y Blaise G Postoperative cognitive dysfunction after cardiac surgery Chest 2005 128 3664 70 10.1378/chest.128.5.3664 16304328

[B43] Mehta Y Singh R Cognitive dysfunction after cardiac surgery J Alzheimers Dis 2010 22 Suppl 3 115 20 20858972 10.3233/JAD-2010-100834

[B44] Dong Y Zhang G Zhang B Moir RD Xia W Marcantonio ER Culley DJ Crosby G Tanzi RE Xie Z The common inhalational anesthetic sevoflurane induces apoptosis and increases beta-amyloid protein levels Arch Neurol 2009 66 620 31 10.1001/archneurol.2009.48 19433662 PMC2748878

[B45] Xie Z Dong Y Maeda U Moir R Inouye SK Inouye SK Culley DJ Crosby G Tanzi RE Isoflurane-induced apoptosis: a potential pathogenic link between delirium and dementia J Gerontol A Biol Sci Med Sci 2006 61 1300 6 10.1093/gerona/61.12.1300 17234824

[B46] Xie Z Dong Y Maeda U Moir D Xia W Culley DJ Crosby G Tanzi RE The inhalation anesthetic isoflurane induces a vicious cycle of apoptosis and amyloid beta-protein accumulation J Neurosci 2007 27 1247 54 10.1523/JNEUROSCI.5320-06.2007 17287498 PMC6673586

[B47] Zhang B Dong Y Zhang G Moir RD Xia W Yue Y Tian M Culley DG Crosby G Tanzi RE The inhalation anesthetic desflurane induces caspase activation and increases amyloid beta-protein levels under hypoxic conditions J Biol Chem 2008 283 11866 75 10.1074/jbc.M800199200 18326038 PMC2335348

[B48] Rocca WA Fratiglioni L Bracco L Pedone D Groppi C Schoenberg BS The use of surrogate respondents to obtain questionnaire data in case-control studies of neurologic diseases J Chron Dis 1986 39 907 12 10.1016/0021-9681(86)90039-1 3793841

