# Supplementary material for: Exposure to general anesthesia and risk of alzheimer's disease: a systematic review and meta-analysis
Source: BMC Geriatr. 2011 Dec 14;11:83. doi: 10.1186/1471-2318-11-83 (PMC3258207; doi:10.1186/1471-2318-11-83)
Supplement: Additional file 1 — Document 1: Electronic Database Search Strategy. [file 1471-2318-11-83-S1.DOC]

**Document 1**: Electronic Database Search Strategy

MEDLINE (1980 - April 27, 2010):

1. exp Alzheimer Disease/ OR alzheimer*.mp. OR dement*.mp. OR exp Dementia/

2. exp Anesthetics/ or exp Anesthesia/ or (anesthe* or anaesthe*).mp. or exp Surgical Procedures, Operative/ or surgery.mp. or exp Postoperative Complications/

3. cohort studies/ or longitudinal studies/ or follow-up studies/ OR prospective studies/ OR case-control studies/ or retrospective studies/ or cross-sectional studies/ OR risk/ or logistic models/ or risk assessment/ or risk factors/ OR causality/ or precipitating factors/ or risk factors/ OR risk.tw. OR (cause or causal or causation).tw.exp OR relative risk.tw.

Embase (1980 - April 27,2010)

1. exp Alzheimer Disease/ OR Alzheimer* OR dement*

2. exp Anesthetics/ OR exp anesthesia/ OR (anesthe* or anaesthe*).mp OR surgery.mp OR surgical procedures.mp OR exp postoperative complication/

3. cohort studies/ or longitudinal studies/ or follow-up studies/ or prospective studies/ or case-control studies/ or retrospective studies/ or cross-sectional studies/ or risk/ or logistic models/ or risk assessment/ or risk factors/ or causality/ or precipitating factors/ or risk factors/ or risk.tw. or (cause or causal or causation).tw. or odds ratio.tw. or relative risk.tw.
