# Supplementary material for: Exposure to general anesthesia and risk of alzheimer's disease: a systematic review and meta-analysis
Source: BMC Geriatr. 2011 Dec 14;11:83. doi: 10.1186/1471-2318-11-83 (PMC3258207; doi:10.1186/1471-2318-11-83)
Supplement: Additional file 2 — Document 2: Risk of Bias Assessment Tool - Adapted from the Newcastle-Ottawa Risk of Bias Assessment for Case-Control Studies. [file 1471-2318-11-83-S2.DOCX]

**Document 2:** Risk of Bias Assessment Tool – Adapted from the Newcastle-Ottawa Risk of Bias Assessment for Case-Control Studies.

|  | **Methods for Defining Cases** | **Selection of Cases** | **Methods for Defining Controls** | **Selection of Controls** | **Comparability of Cases and Controls** | **Similar Methods for Defining Exposure** | **Non-Response Bias** |
| --- | --- | --- | --- | --- | --- | --- | --- |
| **Low Risk of Bias** | - Standardized criteria for dementia - Criteria described in sufficient detail | - Community based sample of cases | - Same methods used to define cases | - Community based sample for controls | - Matched or adjusted for a minimum of age and gender | - Proxy reporters used to define exposure in cases and controls or similar methods | - Similar for both groups |
| **High Risk of Bias** | - Criteria for diagnosis dementia unclear | - Hospital, clinic or other source for cases | - Different methods used to define cases and controls | - Clinic, hospital or non-random community based selection of controls | - Not matched or adjusted for age and gender | - Proxy report only for cases or methods of determining exposure different for cases and controls | - Different for both groups or non-respondents described |
