# Supplementary material for: Exposure to general anesthesia and risk of alzheimer's disease: a systematic review and meta-analysis
Source: BMC Geriatr. 2011 Dec 14;11:83. doi: 10.1186/1471-2318-11-83 (PMC3258207; doi:10.1186/1471-2318-11-83)
Supplement: Additional file 3 — Table S1: Case-Control Studies Examining Association between General Anesthesia and Alzheimer's Disease. [file 1471-2318-11-83-S3.DOCX]

**Table 1:** Case-Control Studies Examining Association between General Anesthesia and Alzheimer’s Disease

| **Study Name** | **Total Number** | **Mean Age** | **Female Gender, Number (%)** | **Study Sample** | **Exposure Definition** | **Outcome Definition** | **Matching Variables** | **Adjustment** | **Proxy Reporters** | **Comments** |
| --- | --- | --- | --- | --- | --- | --- | --- | --- | --- | --- |
| Heyman, 1984[24]  Cases  Controls | 40  80 | 60.8 | -- | Inpatients  Community | Surgery with GA | Clinical criteria | Age (5 years), gender, race | -- | Family member |  |
| French, 1985[25]  Cases  Controls (H)*  Controls (C) | 78  76  48 |  | 0 (0) | Clinic based  Hospital  Community | Any previous exposure to GA | Clinical diagnosis | Age (2 years), gender, race | -- | “Usually next of kin” | All participants were male |
| Amaducci, 1986[26]  Cases  Controls (C)*  Controls (H) | 116  116  97 | -- | 75 (64.6) | Clinic | Any history of GA | Clinical diagnosis | Age (3 years), gender | -- | Next of kin | Cases provided names controls |
| Broe,1990[27]  Cases  Controls | 170  170 | 78  78 | 106 (62.3)  106 (62.3) | Clinics  Clinics | Any GA | NINDS-ADRDA | Age (2 years), gender | -- | Informant |  |
| Graves, 1990[28]  Cases  Controls | 130  130 | 66·2  63·6 | 60 (46.2)  60 (46.2) | Clinic  Community | Any history of GA | DSM-IV | Age (10 years), gender | -- | Spouse or surrogate | Cases provided names of controls |
| Kokmen, 1991[29]  Cases  Controls | 415  415 | -- | -- | Clinic based | Any prior GA in medical record | Clinical criteria | Age (3 years), gender | -- | -- |  |
| Li, 1992[30]  Cases  Controls | 70  140 | 65·2  65·4 | 37 (52.8)  74 (52.8) | Clinic/hospital  Community | Any GA | NINDS-ADRDA | Age (3 years), gender | -- | Family member |  |
| Bohnen, 1994[31]  Cases  Controls | 252  252 | --  -- | 205 (81)  205 (81) | Clinic sample | Any GA recorded in medical record | According to algorithm and criteria | Age (1 year), gender | -- | -- |  |
| CSHA, 1994[32]  Cases  Controls | 224  529 | 84·7  79·0 | -- | Community  Community | Any GA | DSM-IV,  NINDS-ADRDA |  | Age, gender, education, LTC status | Proxy reporters |  |
| Tyas, 2001[33]  Cases  Controls | 36  658 | 79·8  73·7 | 24 (66.7)  409 (62.2) | Community  Community | Any GA | NINDS-ADRDA | -- | Age, gender, education | Cases interviewed prior to development of dementia | Incident cases of dementia |
| Gasparini, 2002[34]  Cases  Controls (PD)  Controls (Other)* | 115  230  230 | 69·1  69·7  68·9 | 78 (67.8)  156 (67.8)  156 (67.8) | Clinic  Clinic  Clinic | Any GA in medical records | NINDS-ADRD | Age (3 years), gender |  | -- | Exposure in 5 years preceding outcome |
| Harmanci, 2003[35]^41^  Cases  Controls | 57  127 | 77·3  76·1 | 41 (71.2)  79 (62.2) | Community  Community | Any history of GA | DSM-IV TR  NINDS-ADRDA | -- | Stepwise logistic regression | Spouse or other family members |  |
| Yip, 2006[36]  Cases  Controls | 133  2453 | -- | 90 (67.6)  1542 (62.8) | Community  Community | Any GA | AGECAT criteria | -- | Age, gender, social class, education | Cases were interviewed prior to dementia | Incident cases of dementia in cohort study |
| Plassman, 2009[37]  Cases  Controls | 260  288 | 82·7  78·6 | 136 (52.4)  193 (66.9) | Community  Community | Any GA in administrative databases | Comprehensive assessment | -- | Age, gender, education, race | -- |  |
| Zuo, 2010[38]  Cases  Controls | 26  52 | 76  76 | 13 (50)  25 (48) | Patients who received spine surgery | Any GA in administrative database | ICD 10 codes for dementia | Age (1 year) | Stepwise logistic regression | -- |  |

*Control group used for meta-analysis; AGECAT=Automated Geriatric Examination for Computer Assisted Taxonomy; C=community based controls; DSM-IV=Diagnostics and Statistic Manual of Mental Disorders; GA= general anesthesia; H=hospital- based controls; LTC=long-term care; NINDS-ADRDA= National Institutes of Neurological and Communicative Disorders and Stroke – Alzheimer’s Disease and Related Disorders; PD=Parkinson’s disease, Other = other neurological disorder
