# Supplementary material for: Exposure to general anesthesia and risk of alzheimer's disease: a systematic review and meta-analysis
Source: BMC Geriatr. 2011 Dec 14;11:83. doi: 10.1186/1471-2318-11-83 (PMC3258207; doi:10.1186/1471-2318-11-83)
Supplement: Additional file 4 — Table S2: Association between Exposure to General Anesthesia and Alzheimer's Disease in Case-Control Studies. [file 1471-2318-11-83-S4.DOCX]

**Table 2:** Association between Exposure to General Anesthesia and Alzheimer’s Disease in Case-

Control Studies

|  | **Number Exposed** | **Total Number** | **Odds Ratio**  **(95% CI)** |
| --- | --- | --- | --- |
| Heyman, 1984[24]  Cases  Controls | 34  72 | 40  80 | 0.63 (0.20 – 1.96) ^†^ |
| French, 1985[25]  Cases  Controls | --  -- | 78  76 | 2.00 (0.32 – 15.76)^*^ |
| Amaducci, 1986[26]  Cases  Controls | 28  27 | 32  32 | 1.30 (0.31 – 5.35)^*^ |
| Broe, 1990[27]  Cases  Control | 147  148 | 170  170 | 0.95 (0.50 - 1.81)^*^ |
| Graves, 1990[28]  Cases  Controls | 104  105 | 130  130 | 1.21 (0.54 – 2.73)^+^ |
| Kokmen,1991[29]  Cases  Controls | 285  295 | 415  415 | 0.86 (0.6 – 1.21)^+^ |
| Li, 1992[30]  Cases  Controls | 5  13 | 66  132 | 0.62 (0.20-1.86)^*^ |
| Bohnen, 1994[31]  Cases  Control | 208  199 | 252  252 | 1.26 (0.81 – 1.96) ^†^ |
| CSHA, 1994[32]  Cases  Controls | 176  420 | 204  480 | 1.07 (0.60 – 1.90)^+^ |
| Tyas, 2001[33]  Cases  Controls | 28  564 | 34  632 | 0.61 (0.22 – 1.63)^+^ |
| Gasparini, 2002[34]  Cases  Controls | 89  190 | 115  230 | 1.03 (0.60 – 1.75)^†^ |
| Harmanci, 2003[35]  Cases  Controls | 35  78 | 57  127 | 1.2 (0.58 – 2.48)^+^ |
| Yip, 2006[36]  Cases  Controls | 92  1947 | 133  2453 | 0.7 (0.4 – 1.1)^+^ |
| Plassman,2009[37]  Cases  Controls | -- | -- | 1.21 (1.00 - 1.41)^+^ |
| Zuo, 2010[38]  Cases  Controls | 18  38 | 26  52 | 0.83 (0.29 – 2.33) ^†^ |

^*^OR from matched analysis reported in study;^+^ odds ratio from adjusted analysis reported in study; ^†^odds ratio calculated from numbers of exposed cases and controls
