# Supplementary material for: Exposure to general anesthesia and risk of alzheimer's disease: a systematic review and meta-analysis
Source: BMC Geriatr. 2011 Dec 14;11:83. doi: 10.1186/1471-2318-11-83 (PMC3258207; doi:10.1186/1471-2318-11-83)
Supplement: Additional file 5 — Table S3: Assessment of Risk of Bias in Included Studies. [file 1471-2318-11-83-S5.DOCX]

**Table 3:** Assessment of Risk of Bias in Included Studies

| **Study Name** | **Case Definition** | **Selection of Cases** | **Control Definition** | **Selection of Controls** | **Comparability of Cases and Controls** | **Methods for Determining Exposure** | **Same Methods for Cases and Controls** | **Overall^a^** |
| --- | --- | --- | --- | --- | --- | --- | --- | --- |
| Heyman, 1984[24] | Low | High | High | Low | High | Low | Low | High |
| French, 1985[25] | High | High | High | High | Low | Low | Unclear | High |
| Amaducci, 1986[26] | Low | High | High | High | Low | Low | Low | High |
| Broe,1990[27] | Low | High | High | High | Low | Low | High | High |
| Graves, 1990[28] | Low | High | Low | High | Low | Low | Low | High |
| Kokmen, 1991[29] | High | High | High | High | Low | Low | Low | High |
| Li,1992[30] | Low | High | High | Low | Low | Low | Low | High |
| Bohnen, 1994[31] | Low | High | Low | High | Low | High | Low | High |
| CSHA, 1994[32] | Low | Low | Low | Low | Low | Low | Low | Low |
| Tyas,2001[33] | Low | Low | Low | Low | Low | Low | Low | Low |
| Gasparini,2002[34] | Low | High | High | High | Low | Low | Low | High |
| Harmanci, 2003[35] | Low | Low | Low | Low | High | Low | Low | High |
| Yip, 2006[36] | Low | Low | Low | Low | Low | Low | Low | Low |
| Plassman,2009[37] | Low | Low | Low | Low | Low | Low | Low | Low |
| Zuo, 2010[38] | High | High | High | High | Unclear | Low | Low | High |

**Note:** Items rated as high indicate that the study is at high risk of bias for that particular item

a. Overall risk of bias was rated as low if case definition, selection of cases and controls, and comparability of cases and controls were all rated as low risk of bias.
